# Motor skills and cognitive benefits in children and adolescents: Relationship, mechanism and perspectives

**DOI:** 10.3389/fpsyg.2022.1017825

**Published:** 2022-11-21

**Authors:** Peng Shi, Xiaosu Feng

**Affiliations:** ^1^School of Physical Education, Shanghai University of Sport, Shanghai, China; ^2^Physical Education College, Liaoning Normal University, Dalian, China

**Keywords:** motor skills, cognition, brain science, children and adolescents, executive function

## Abstract

**Objective:**

There is a strong interaction between motor skills and cognitive benefits for children and young people. The aim of this paper is to explore the relationship between motor skill types and their development and the cognitive benefits of children and adolescents. In turn, on this basis, it proposes pathways and mechanisms by which motor skills improve cognition, and provide a basis for subsequent teaching of skills that follow the laws of brain cognitive development.

**Methods:**

This paper summarizes the research on the relationship between different types of motor skills and their development and cognitive benefits of children and adolescents. Based on these relationships, pathways, and mechanisms for motor skills to improve cognition are tentatively proposed.

**Results:**

There is an overall pattern of “open > closed, strategy > interception, sequence > continuous” between motor skill types and the cognitive benefits of children and adolescents. Long-term motor skill learning practice is accompanied by increased cognitive benefits as skill proficiency increases. The dynamic interaction between motor skills and physical activity exposes children and adolescents to environmental stimuli and interpersonal interactions of varying complexity, promoting the development of agility, coordination and cardiorespiratory fitness, enhancing their motor experience, which in turn improves brain structure and functional activity.

**Conclusion:**

Motor skills training promote cognitive efficiency in children and adolescents. Motor skill interventions that are open-ended, strategic and sequential in nature are more effective. Environmental stimuli, interpersonal interaction, agility, coordination, and cardiorespiratory fitness can be considered as skill attribute moderators of motor skills to improve cognition.

## Introduction

Cognition refers to the process in which individuals extract, process, and store information (Bayne et al., 2019). It is the representation of the interconnection and interaction between objective things (Ahmed et al., 2020). Cognition usually includes perception, attention, memory, thinking, and imagination (Shao, 2019). It is an important indicator to measure individual survival and development. In particular, children and adolescents are the golden age of cognitive development. The level of cognitive development at this stage is of great significance to individual academic performance, mental health, and social development in adulthood (Blakemore and Choudhury, 2006; Ahmed et al., 2020). For example, fluid intelligence and executive function are closely related to the academic performance and acquired achievement of children and adolescents (Graham et al., 2017; Tikhomirova et al., 2020). For another example, lower executive control is associated with aggressive behavior, suicidal tendencies, anxiety and depression, Internet addiction (Nordahl et al., 2019; Hu J. et al., 2022). Therefore, studying the cognitive development and enhancement strategies of children and adolescents is not only a prerequisite for their healthy physical and mental, but also an important guarantee for building an “Intellectual Superpower” and a “Healthy China.”

The relationship between physical activity and cognitive benefit has received increasing attention. Physical activity can improve the structural plasticity of gray matter and white matter of children and adolescents (Xiong et al., 2018; Migueles et al., 2020), promote the change of brain activation pattern under specific tasks (Chaddock-Heyman et al., 2013), improve brain structure and functional networks (Chen et al., 2016), and then promote the improvement of cognitive benefits such as attention (Moratal et al., 2020), memory (Roig et al., 2013), thinking (Ballester et al., 2018), and executive function (Xue et al., 2019) in children and adolescents. With the continuous accumulation of research, researchers have gradually paid attention to the discussion on the dose-effect relationship between physical activity and cognitive benefits of children and adolescents. Most studies focus on the quantitative characteristics (intensity, period, frequency, and duration) of physical activity. A review by Chen et al. (2021) showed that moderate intensity aerobic exercise for more than 30 min has the best effect on the executive function of children and adolescents. Zhou and Jin (2021) summarized that moderate physical activity lasting at least 3 days a week, at least 60 min a day are most conducive to improving the brain function of children and adolescents.

At present, some researchers call for attention to the qualitative characteristics (e.g., energy metabolism, skill types, peer participation, etc.) of physical activity (Tomporowski et al., 2015; Diamond and Ling, 2016). Among them, motor skills are essential for human physical activity and survival and development. On the one hand, the repetitive exercise of multi joint muscles with rich cognitive participation activates the relevant neural circuits (Diamond, 2015; Lakhani et al., 2016). On the other hand, the stimulation of changes in the sports environment requires more individual participation in the decision-making process, and changes brain function and brain network under the interaction of individual, environment and behavior (Nithianantharajah and Hannan, 2006; Sale et al., 2009; Tomporowski and Pesce, 2019). Zhou and Jin (2021) believed that motor skills have a specialization attribute on the cognitive benefits of children and adolescents. So, what are the characteristics of this specialized attribute? That is, what are the pathways and moderators by which motor skills promote cognitive benefits in children and adolescents? In addition, cognitive brain function develops to regulate the learning and control of motor skills. The dorsolateral prefrontal cortex, a brain-activated region of executive function, is closely linked to the motor system, and has an important role in motor sequence learning and monitoring behavioral execution (Kraeutner et al., 2016). Selective attention and spatial working memory capacity are important for stimulus discrimination, response selection and response programming during motor decision making (Wang, 2013). Therefore, it is possible to explore the pathways and moderators of motor skills to improve the cognition of children and adolescents, and then incorporate these moderators into motor skills teaching practices, thereby achieving joint improvements in motor skills and cognitive function. The purpose of this review is to review relevant study on the relationship between different types of motor skills and cognitive benefits in children and adolescents, and to explore the pathways, mechanisms and moderators of motor skills to improve cognition. On this basis, future study is envisaged, so as to inform subsequent research and teaching practice.

## Cross sectional studies on motor skills and cognitive benefits

It has become a widespread consensus that physical activity promote the cognitive benefits of children and adolescents (Fedewa and Ahn, 2011; García-Hermoso et al., 2021). At present, researchers are gradually concerned about which kind of motor skills have greater cognitive benefits (Gu et al., 2019). According to the predictability of environmental changes, motor skills can be divided into open skills and closed skills (Zhang, 2012). Changes in the open skills environment are unpredictable, and the quality of cognitive decision-making is the main determinant of success; the closed skills environment is predictable, and the quality of action control is the main determinant of success (Zhang, 2012). A study on athletes showed that athletes who have long been engaged in open skills training show more cognitive advantages in general cognitive tasks than athletes who have been engaged in closed skills training (Wang et al., 2016). Yu et al. (2017, 2019) compared the performance of proactive and reactive control between badminton (open skill) and track and field (closed skill) athletes, and found that badminton athletes have a higher active control performance. Krenn et al. (2018) further divided open skills into interception skills (e.g., badminton, tennis, etc.) and strategic skills (e.g., football, ice hockey, etc.), and compared the executive function performance of interception, strategic and closed skill athletes. The results showed that compared with closed skill athletes, strategic skill athletes showed unique cognitive advantages in inhibitory control, working memory, cognitive flexibility, and interception skill athletes only performed well in inhibitory control. However, a meta-analysis (Voss et al., 2010) showed that interception skill athletes have better cognitive performance than strategic skill athletes. Differences in the motor environment and the cognitive demands of interception and strategic skills may result in different changes in brain organization. Badminton players (interception skill) have significantly increased gray matter volumes in the left inferior frontal gyrus, left superior parietal lobule, and left precuneus (Wu et al., 2015). These brain structures are associated with fine motor control and spatial position perception (Macuga and Frey, 2014). Basketball players (strategic skill) have significantly increased gray matter volumes in the infratemporal gyrus, left middle frontal gyrus, left inferior frontal gyrus, middle cingulate gyrus, and insula (Wu et al., 2015). These brain structures are related to visual information processing, response inhibition control, and perceptual motor decision-making (Simmons et al., 2012).

Similarly, different types of motor skills have different associations with cognitive performance in children and adolescents. Zhang et al. (2009) used event-related potentials to compare the degree of brain activation in GO/NO-GO tasks in children involved in table tennis and swimming training. The results found that the table tennis group had higher task accuracy and lower N2 amplitude compared to the swimming group. A study (Ji, 2014) compared the relationship between four open skills and executive function in pupils. The results found that the basketball and table tennis groups had the best inhibitory control; the badminton and taekwondo groups had the best cognitive flexibility. However, cross-sectional studies still need to consider more confounding factors (e.g., physical activity (Roig et al., 2013; Ballester et al., 2018; Xue et al., 2019; Moratal et al., 2020), socio-economic status (Duncan et al., 2017), sleep (Turnbull et al., 2013), etc.), in order to more accurately describe the relationship between motor skills and executive function. In addition, cross-sectional studies cannot elucidate the causal relationship between the two, so a series of longitudinal intervention studies are necessary to explore this.

## Interventional studies on motor skills and cognitive benefits

The advantages of real-word settings are that the equipment requirements are simpler, and easier to integrate into school physical education classes or extra-curricular sports activities (Zhao et al., 2015). Therefore, this study systematically searched for studies of exercise interventions for cognitive function in children and adolescents in real-word settings, excluding intervention studies of treadmill or bicycle ergometer in laboratory settings. The focus of this study was to sort out the effects of acute or long-term interventions in different types of motor skills on the cognition of children and adolescents. This study focuses on sorting out the effects of acute and long-term interventions for different types of motor skills. Acute interventions are those in which the individual receives a brief or one-off exercise session; long-term interventions are those in which the individual receives a longer period of exercise (De Greeff et al., 2018; Zhong et al., 2022).

### Effects of acute interventions in motor skills on cognition

Exercise has immediate cognitive effect, and a single exercise intervention can produce certain cognitive benefits (Tomporowski, 2003). This study reviewed research on the cognitive effects of acute interventions of motor skills in children and adolescents (Table 1), and found that open-sequence skills, which constitute cognitive challenge and physical coordination, promoted attention (Gallotta et al., 2015) and verbal working memory (O’Brien et al., 2021) better than closed-sequence skills in children and adolescents. In addition, Manion and Alexander (1997) suggest that working on tasks with peers improves children’s executive functions such as strategy selection and application, and problem understanding and solving. Chen et al. (2015b) increased the unpredictability of the sport environment through peer cooperative rope skipping, further improving the effectiveness of the intervention on executive function. Ottoboni et al. (2021) compared the effects of balls (sequence skill) and obstacle running (continuous skill) with the same open attribute on verbal and visual spatial working memory in children aged 7 to 10 years and found better results for open-sequence skills. Yan et al. (2014) compared the intervention effects of obstacle running (open-continuous) and aerobics (closed-sequence) on the executive function of primary school students, and showed that there was a selective facilitation of both, without reflecting an absolute advantage of open or sequence skills. The reason for this is the interaction between the action structure (sequence and continuous) and the environmental context (open and closed). In summary, the effects of exercise interventions on children’s and adolescents’ cognition show a pattern of open skills over closed skills and sequence skills over continuous skills.

**Table 1:** Comparative study of acute interventions of motor skills on the cognition of children and adolescents.

Included articles study design	Patients (*N*/Age)	Interventions and controls	Outcome	
			Outcome measures	Results
Gallotta et al. (2015)	E1 = 31/8 ~ 11y	50 min traditional PE course (brisk walking, jogging, jumping, etc.) (E1) vs. basketball skills acquisition practice (E2) vs. basic academic course (C)	①d2-test (E1>C>E2)	+&
RCT	E2 = 46/8 ~ 11y			
	C = 39/8 ~ 11y			
Yan et al. (2014)	E1 = 52/9.8 ± 0.3y	30 min moderate intensity (60–69% HRmax) aerobics (E1) vs. obstacle run (E2) vs. sitting (C)	②Flanker (E1>E2)	+&
RCT	E2 = 51/9.7 ± 0.3y		③1-back (E1>E2)	+&
	C = 51/9.8 ± 0.3y		④More-odd shifting (E2>E1)	+&
Chen et al. (2014)	E1 = 30/9.8 ± 0.3y	30 min low intensity (50–59% HRmax) basketball high dribbling and dribbling between runs (E1) vs. moderate intensity (60–69% HRmax) (E2) vs. high intensity (70–79% HRmax) (E3) vs. free activities in their classroom (C)	②Flanker (E2>E1 = E3>C)	+&
RCT	E2 = 30/9.8 ± 0.3y		③1-back (E2 = E3>E1 = C)	+0&
	E3 = 32/9.7 ± 0.3y		④More-odd shifting (E2>E3 = C>E1)	+0&
	C = 28/9.8 ± 0.3y			
Chen et al. (2015a)	E1 = 22/9.3 ± 0.3y	8 min (E1), 15 min (E2), 30 min (E3) moderate intensity (60–69% HRmax) basketball high dribbling and dribbling between runs vs. 8 min (T1), 15 min (T2), 30 min (T3) free activities in their classroom	②Flanker (E1 = C1; E2>C2; E3>C3)	0
RCT	E2 = 22/9.4 ± 0.3y		③1-back (E1 = C1; E2 = C2; E3>C3)	0
	E3 = 24/9.5 ± 0.3y		④More-odd shifting (E1 = C1; E2 = C2; E3>C3)	0
	C1 = 20/9.4 ± 0.3y			
	C2 = 20/9.4 ± 0.3y			
	C3 = 22/9.5 ± 0.3y			
Chen et al. (2015b)	E1 = 39/9.1 ± 0.3y	30 min moderate intensity (60–69% HRmax) cooperative rope skipping (E1) vs. single rope skipping (E2) vs. sedentary reading (C)	②Flanker (E1>E2)	+&
RCT	E2 = 38/9.1 ± 0.3y		③1-back (E1>E2)	+&
	C = 38/9.2 ± 0.4y		④More-odd shifting (E1>E2)	+&
O’Brien et al. (2021)	E1 = 16/7.0 ± 0.5y	30 min open skills activities such as basketball, football, tennis (E1) vs. closed skills activities such as race, rope skipping, circuit training (E2) vs. free activities in their classroom (C)	⑤Corsi blocks test	0
RCT	E2 = 16/6.7 ± 0.1y		⑥Backward Digit Span (E1>E2)	+&
	C = 19/7.0 ± 0.5y			
Ottoboni et al. (2021)	125/7 ~ 10y	30 min high intensity (170 ~ 180 bpm) team ball games (E1) vs. agility obstacle run (E2) vs. basic academic course (C)	⑤Corsi blocks test (E1>E2)	+&
RCT			⑥Digit Span (E1>E2)	+&

RCT, randomized controlled trial; E, experimental group; C, control group; y, year; HRmax, maximum heart rate; ①, attention; ②, inhibitory control; ③, working memory shifting; ④, cognitive flexibility; ⑤, visual spatial working memory; ⑥, verbal working memory; +, beneficial to experimental group; −, beneficial to control group; 0, no significant difference between the experimental and control groups; &, comparison of intervention results between experimental groups.

In addition, some studies have examined the effects of acute interventions for motor skills of varying intensity and duration on the cognition of children and adolescents. Studies have shown an “inverted U-shaped” dose–response relationship between exercise intensity and cognitive performance, with moderate intensity being more beneficial to the development of executive function (Chang et al., 2011; Ludyga et al., 2016). Transient low activation theory (Dietrich, 2003) believes that human brain resources are limited. The motor system in high-intensity exercise requires more metabolic resources to control body movements and maintain motor performance, while relatively fewer resources are available for cognitive processing, which may impair cognitive performance (Dietrich, 2003; Browne et al., 2017). However, Chen et al. (2014) compared the effects of acute interventions with different intensities of basketball dribbling, and found that moderate intensity exercise was the most effective for executive function in primary school children, but that high intensity exercise helped to improve inhibition and refreshment functions as well. The evidence of sports experience showed that the learning and training process of complex motor skills can improve the peer relationship of children and adolescents, and it is easier to stimulate their exercise enjoyment and positive emotional experience. Some studies (Zhang et al., 2019; Herbert et al., 2020) have shown that lower exercise enjoyment and social motivation are more likely to contribute to fatigue during exercise. Therefore, motor skills may interact with exercise intensity, but there is no study involved at present. Chen et al. (2015a) compared the effects of acute interventions of varying durations of moderate intensity basketball dribbling on the executive function of primary school students, and found that 30 min was the most effective intervention. However, no studies have investigated the time-course effects of acute intervention of complex motor skills on the cognitive function of children and adolescents, and it is unknown how long the cognitive benefits can be maintained after the intervention.

### Effects of long-term interventions in motor skills on cognition

This study reviewed research on the long-term intervention effects of motor skills on children’s and adolescents’ cognition (Table 2), and found that different types of motor skills improved children’s and adolescent’s cognitive functions to a certain extent, and open skills and sequence skills had better effects on attention (Kong, 2012), executive function (Schmidt et al., 2015) and verbal working memory (Koutsandreou et al., 2016). Tse et al. (2021) similarly demonstrated a more positive effect of open motor skill learning (learning to ride a bicycle in a natural environment) than closed continuous exercise (riding a stationary bicycle) on the improvement of executive function and visual spatial working memory in children with autism spectrum disorders. However, one study (Telles et al., 2013) showed that continuous skills based on jogging, sprint running and relay races were more effective than yoga (sequential skill) as an intervention for inhibitory control in school-aged children. The reason for this may be that the physical movement changes in yoga are less frequent than sequential skills such as aerobics and fitness boxing, and stimulation of the dorsolateral prefrontal cortex is lower (Koziol and Lutz, 2013). A recent Meta-analysis (Zhang et al., 2022) evaluated the effect of 11 motor skills on working memory in school-aged children, with an overall pattern of “open > closed, sequence > continuous.” In addition, Wu et al. (2007) compared the intervention effects of football (strategic skills) and table tennis (interception skills) on the attention qualities of primary school students. The moderate- and low-intensity football interventions were found to be more effective than table tennis in terms of attention sustainment and attention shifting, respectively; whereas the mederate-intensity table tennis intervention was more effective than football in terms of attention stability, attention breadth and attention concentration. Thus, strategic and interception skills have a selective facilitative effect on the attention qualities of children and adolescents.

**Table 2:** Comparative study of long-term interventions of motor skills on the cognition of children and adolescents.

Included articles study design	Patients (*N*/Age)	Interventions and controls	Outcome	
			Outcome measures	Results
Kong (2012)	E1 = 30/grade 1 and 5	16 weeks (3x/week) moderate intensity (65–75% HRmax) table tennis, 40 min/time (E1) vs. fitness boxing (E2) vs. rope skipping (E3) vs. self-study or book reading (C)	①Digit cancellation test (E1 = E2>E3 = C)	+0&
non-RCCT	E2 = 30/grade 1 and 5			
	E3 = 30/grade 1 and 5			
	C = 30/grade 1 and 5			
Wu et al. (2007)	E1 = 30/grade 5	12 weeks (3x/week) low intensity (105–130 beats/min) football, 35 min/time (E1) vs. moderate intensity (130–150 beats/min) football (E2) vs. low intensity table tennis (E3) vs. moderate intensity table tennis (E4) vs. self-study or reading books (C)	①Attention test	
non-RCCT	E2 = 29/grade 5		Attention stability (T2<T4)	+&
	E3 = 28/grade 5		Attention span (T2<T4)	+&
	E4 = 27/grade 5		Attention shift (T1>T3; T2<T4)	+&
	C = 29/grade 5		Attention duration (T2>T4)	+&
			Attention concentrativeness (T2<T4)	+&

Telles et al. (2013)	E1 = 49/10.4 ± 1.2y	12 weeks (5x/week) yoga, 45 min/time (E1) vs. physical exercise such as jogging, sprint running, relay races (E2) vs. pre-test	②Stroop (E1<E2)	+&
RCT	E2 = 49/10.5 ± 1.3y			
Schmidt et al. (2015)	E1 = 69/11.3 ± 0.6y	6 weeks (2x/week) high intensity soft hockey and basketball games, 45 min/time (E1) vs. 200 m round trip run (E2) vs. traditional PE course (C)	②Flanker (E1>E2 = C)	+0&
RCT	E2 = 57/11.3 ± 0.6y		③2-back (E1>E2 = C)	+0&
	C = 55/11.4 ± 0.6y		④More-odd shifting (E1>E2 = C)	+0&
Koutsandreou et al. (2016)	E1 = 27/9.3 ± 0.6y	10 weeks (3x/week) moderate intensity (60–70% HRmax) aerobic exercise, 45 min/time (E1) vs. moderate intensity (55–65% HRmax) skill practice focused on improving coordination (S) (E2) vs.do their homework (C)	⑥Letter Digit Span (E2>E1)	+&
RCT	E2 = 23/9.4 ± 0.7y			
	C = 21/9.3 ± 0.6y			
Tse et al. (2021)	ASD	2 weeks (5x/week) low intensity (RPE = 3–5) learning to cycle in a natural environment, 60 min/time (E1) vs. ride a stationary bike (E2) vs. daily activity (C)	②GO/NO GO (E1>E2 = C)	+0&
RCT	E1 = 22/10.2 ± 0.7y		⑤Corsi blocks test (E1>E2 = C)	+0&
	E2 = 20/9.6 ± 1.6y		⑥Forwards/Backwards Digit Span (E1 = E2 = C/E1 = C>E2)	-0&
	C = 20/9.9 ± 1.3y		⑦Tower of London (E1>E2 = C)	+0&
	
Pan et al. (2016)	1 = LD; 2 = Normal	10 weeks (3x/week) moderate intensity (60–69% HRmax) basketball intervention, 30 min/time (E) vs. traditional PE course (C)	②Flanker (E1>E2)	+&
RCT	E1 = 23/12.0 ± 0.6y		③1-back (E1>E2)	+&
	E2 = 25/12.0 ± 0.6y		④More-odd shifting (E1>E2)	+&
	C1 = 22/12.0 ± 0.6y			
	C2 = 23/12.0 ± 0.6y			
Yin et al. (2017)	1 = LD, 2 = Normal	16 weeks (3x/week) moderate intensity (60–69% HRmax) basketball intervention, 30 min/time (E) vs. blank control (C)	②Flanker (E1>E2)	+&
RCT	E1 = 26/grade 4		③1-back (E1 = E2)	+&
	E2 = 26/grade 4		④More-odd shifting (E1>E2)	+&
	C1 = 23/grade 4			
	C2 = 21/grade 4			
Song et al. (2020)	1 = ADHD, 2 = Normal	10 weeks moderate intensity (60–69% HRmax) orienteering, 25-35 min/time (E) vs. traditional PE course (C)	②Flanker (E1>E2)	+&
RCT	E1 = 22/NC		③1-back (E1>E2)	+&
	E2 = 20/NC		④More-odd shifting (E1>E2)	+&
	C1 = 22/NC			
	C2 = 20/NC			

RCT, randomized controlled trial; non-RCCT, non-randomized concurrent control trial; NC, not clear; E, experimental group; C, control group; y, year; HRmax, maximum heart rate; RPE, rating of perceived exertion; ①, attention; ②, inhibitory control; ③, working memory shifting; ④, cognitive flexibility; ⑤, visual spatial working memory; ⑥, verbal working memory; ⑦, planning and problem solving; +, beneficial to experimental group; −, beneficial to control group; 0, no significant difference between the experimental and control groups; &, comparison of intervention results between experimental groups; ADHD, attention deficit hyperactivity disorder; ASD, autism spectrum disorder; LD, learning difficulties.

In addition, studies have demonstrated the effectiveness of interventions in open skills (basketball, orienteering) on executive function in normal pupils and children with cognitive impairment [learning difficulties (Pan et al., 2016; Yin et al., 2017), and attention deficit hyperactivity disorder (Song et al., 2020). The results found that the above exercise interventions were effective for both types of students’ executive function, and the improvement in executive function was higher in children with cognitive impairment. The cognitive load of open skills meets the cognitive memory capacity of children with cognitive impairment, and requires children to switch body movements, inhibit dominant responses and refresh memory information during movement, which can effectively improve executive function and inattention (Song et al., 2020). Also, because of the developmental differences between children with cognitive impairment and normal children, the former have a greater potential for cognitive improvement. Therefore, it is more effective for children with cognitive impairment. In summary, long-term interventions in both open and sequence skills help to improve the cognitive benefits of children and adolescents, particularly in children with cognitive impairments in executive function. However, it is not clear how long this positive intervention effect can be maintained after the long-term intervention for complex motor skills has ended.

## Motor skill proficiency and cognitive benefits

Motor skills play an important role in the life course of individuals. It is one of the internal mechanisms that affect the participation and persistence of physical activity (Laukkanen et al., 2014; Veldman et al., 2018). It interacts with physical activity to jointly maintain the physical health of children and adolescents (Stodden et al., 2008). Based on this, a number of policies in China put forward the goal of making teenagers “proficient in 1 ~ 2 sports skills” (National Health Commission of the People’s Republic of China, 2019; Ministry of Education of the People’s Republic of China, 2021). So, what is the relationship between motor skill proficiency and cognitive benefits?

Brain plasticity refers to the positive changes in the physiological structure and function of molecules, synapses, and cells in the central nervous system of the brain (Sagi et al., 2012). Acute or long-term motor skill learning can cause brain plasticity changes, promote brain angiogenesis, gliogenesis and synaptogenesis, and improve brain structural morphology and functional activities (Zatorre et al., 2012; Vints et al., 2022). Neuroimaging evidence suggests that elite athletes benefit from action experience accumulated through long-term motor skill learning, and tend to increase activity in brain regions (frontal, parietal, occipital, etc.) associated with cognitive understanding (Jacini et al., 2009; Huang et al., 2015; Yang et al., 2020). A meta-analysis by Voss et al. (2010) showed that proficiency in motor skill acquisition was associated with high levels of performance on visual attention processing tests, and that individuals with higher motor skills had greater inhibitory control over distracting stimuli during multi-target tracking. Bi et al. (2020) and Shi et al. (2020) also found that elite athletes who have received professional training have quick response and good accuracy in performing functional tasks. Therefore, it can be shown that long-term motor skill learning and training is accompanied by improved cognitive benefits as it increases skill proficiency.

So, are such results in the field of competitive sports reflected in the children and adolescents? The aforementioned studies have shown that after a period of motor skill learning, cognitive functions such as attention, executive function and working memory improve in children and adolescents with normal and cognitive impairments. This supports, to some extent, the idea that motor skill proficiency is associated with cognitive benefits in children and adolescents. Although the motor skills of the children and adolescents improved after the intervention, it is not clear to what extent motor skills were achieved after the intervention, so it is difficult to draw correlations between motor experience (motor control and perceptual decision-making) and cognitive benefits. Motor skill is a combination of mental and operational processes (Ji et al., 2010). Although motor skills are varied in type and difficulty, whenever they are learned and practiced they necessarily involve the participation of cognitive processes. Motor skill learning processes share brain area activation with cognitive tasks such as executive function in the prefrontal cortex region (Cao et al., 2017). And this region is a key region in the regulation of cognition (Funahashi, 2001; Müller and Knight, 2006). Therefore, long-term sports skill training and competition promotes the development of individual sports cognition, and promotes the development of general cognitive function through transfer. Relevant cross-sectional studies (Hou, 2008; Haapala, 2013; Cadoret et al., 2018) show that higher motor skills are associated with more effective attention, inhibitory control and working memory of children and adolescents. Verburgh et al. (2016) and Moratal et al. (2020) found that children aged 8–12 years who regularly participated in football training had more significant advantages in working memory, attention and information processing speed. Ishihara et al. (2017a,b) evaluated the relationship between tennis frequency and executive function of children and adolescents. After controlling for age, gender, BMI and tennis experience, more frequent tennis is related to higher processing speed and inhibition control of boys, and better working memory of students. Similarly, cross-sectional studies are prone to be affected by confounding factors such as physical activity, socio-economic status, and sleep, which need to be further considered in subsequent studies.

## Pathways and mechanisms for motor skills to enhance cognitive benefits

**Figure 1:**
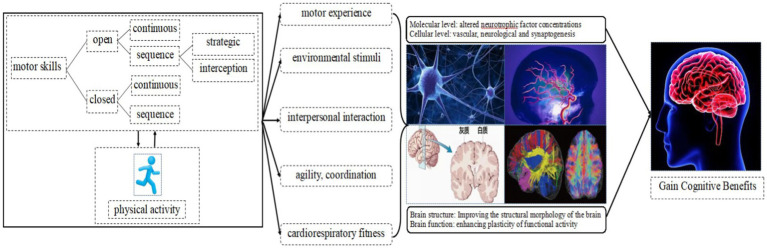
Pathways and mechanisms by which motor skills enhance the cognitive benefits of children and adolescents.

The dynamic mechanism model of physical activity emphasizes that motor skills are not only the internal mechanism that affects the participation and persistence of physical activity, but also interact with physical activity to jointly maintain the physical and mental health benefits of children and adolescents (Stodden et al., 2008). Available evidence (Drollette et al., 2016; Escolano-Pérez and Bestué, 2021) also indicates a direct positive correlation between physical activity and cognition and better academic performance. Based on a review of the relationship between motor skills and the cognitive benefits of children and adolescents, this study proposes pathways and mechanisms by which motor skills enhance the cognitive benefits of children and adolescents (Figure 1). The dynamic interaction between motor skills and physical activity exposes children and adolescents to environmental stimuli and interpersonal interactions of varying complexity, promoting the development of agility, coordination and cardiorespiratory fitness, enhancing their motor experience, which in turn enhances the function of molecules, cells and neural circuits in the nervous system and improves brain structure and functional activity. This is reflected in improvements in attention, executive function, and creative thinking during cognitive tasks.

Increasing the unpredictability of the environmental context, adding variable movement structures and increasing interpersonal interaction processes can increase the complexity and novelty of environmental stimuli, which will promote more physical activity and pose higher cognitive challenges (Nithianantharajah and Hannan, 2006). This rich environmental stimuli increases the concentration of neurotrophins such as brain-derived neurotropic factor and nerve growth factor (Ickes et al., 2000), increases the number of dendritic spines and the volume of synapses on certain neuronal populations (Nithianantharajah and Hannan, 2006), and promotes neuronal activation, signaling, and brain plasticity (Nithianantharajah and Hannan, 2006; Sale et al., 2009). Closed-continuous skills such as jogging and cycling enhance an individual’s cardiorespiratory fitness, increase the capillary density of brain tissue and activate their sensorimotor network, which is the main network regulating response inhibition (Voelcker-Rehage et al., 2011; Shi et al., 2019). Sequence skills have a more complex movement structure and are movement sequences that combine motor coordination and aerobic fitness. The multi-limb involvement and flexibility of movements during the task require more mental manipulation processes and are more likely to induce neurogenesis in the hippocampus, cerebellum, and cerebral cortex (Carey et al., 2005). Open skills such as basketball and table tennis emphasize the combined effects of cardiorespiratory fitness and rich environmental stimuli. This promotes individual perceptual-motor coordination, increases the number of Purkinje neurons and synapses, promotes prefrontal cortical vascularization and induces better neurofunctional remodeling, effectively activating sensorimotor and visual spatial networks (Mavilidi et al., 2015; Moreau et al., 2015).

Rather than emphasizing the neurobiological mechanisms by which motor skills improve cognition, the focus of the pathway places emphasis on the pathways that guide motor skills teaching practice. By distilling the main pathways and cognitive moderators of motor skills to improve the cognition of children and adolescents, one or more of these moderators can be manipulated in subsequent motor skills teaching practices to further promote the cognitive benefits of children and adolescents. Environmental stimuli, interpersonal interaction, agility, coordination, and cardiorespiratory fitness can be considered as skill attribute moderators of motor skills to improve cognition (Figure 1). In addition, there are varying degrees of overlap between these pathways. For example, the complexity of environmental stimuli can be increased through interpersonal interaction; increased environmental stimuli and improved agility, coordination and cardiorespiratory fitness contribute to motor experience. The pathways and mechanisms proposed in this study do not reveal the intrinsic correlations between the pathways, but rather point to actionable variables for teaching motor skills in order to provide guidance for subsequent practice.

### Motor experience

Motor skill proficiency benefits from the movement experience gained during the learning of individual motor skills, including not only the joint-muscle coordination patterns developed during the learning of single technical movements, but also the procedural knowledge gained during the contextualized practice of motor skills. Firstly, according to Pavlov’s theory of conditioned reflexes, motor skills are temporary neural connections established in the relevant cortical centers of the brain with the involvement of multiple sensory organs (Chen et al., 2021). In particular, multiple repetitions of multi-limb, cognitively engaging movements help to activate relevant neural pathways and promote cognitive function (Diamond, 2015; Chen et al., 2021). The rich changes in the external environment stimuli of open skills (e.g., the tearing of offensive and defensive players and the changes in the size of empty spaces, etc.) make individuals constantly face new problems and challenges. Individuals need to coordinate and combine the original actions, coordinate the existing declarative knowledge and experience, make more reasonable coordinated responses or create more novel actions (Kolb and Gibb, 2011; Chen et al., 2021). Therefore, this leads to the continuous connection of neural circuits in the cerebral cortex to improve the motor cognitive process (Kolb and Gibb, 2011; Chen et al., 2021).

### Environmental stimuli

**Figure 2:**
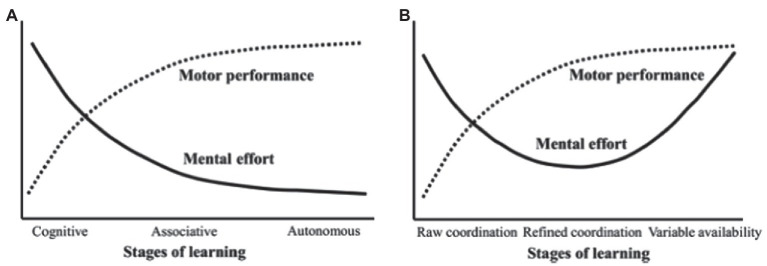
(**A** and **B**) Stages of motor skill acquisition and hypothesized cognitive benefits.

The above studies show that open skills have a greater effect on improving cognitive function in children and adolescents than closed skills. This may be related to the rich environmental stimuli embedded in open skills. The changing and unpredictable sports environment is an ideal place for children and adolescents to develop their cognition (Kolb and Gibb, 2011). Responding and adjusting actions in this sports environment can help to improve motor cognition and motor experience, and then promote the improvement of general cognitive explicit behavior through cognitive transfer (Kolb and Gibb, 2011). Motor skill learning is cognitive and associative in nature. Individuals inhibit irrational visuomotor planning in the early stages and assess new visual stimuli and kinesthetic information through working memory refreshment, activating specific prefrontal areas (Gentili et al., 2013). However, activation in prefrontal areas responsible for inhibition and refreshment decreases as motor skills reach an automated level (Gentili et al., 2013). Motor skill learning consists of the cognitive, associative, and automatic stages. The automatic stage represents the end of the motor skill learning process, where the role of conscious control over the individual’s movements is reduced to a minimum (Figure 2A; Pesce et al., 2019). Therefore, Tomporowski (2003); Tomporowski et al. (2015); Tomporowski and Pesce et al. (2019) proposed to introduce more environmental perturbations in the final stages of the motor skill learning process to generate new cognitive challenges, increasing the flexibility to apply skills in changing environmental stimuli. The complexity and variability of this environmental information provides a continuous stimulus to cognition, which in turn enhances cognitive benefits to a greater extent (Figure 2B; Pesce et al., 2019).

### Interpersonal interaction

Interpersonal interaction is often the primary means of increasing the complexity of exercise situations, and motor skill learning with peer involvement is more conducive to the cognitive benefits for children and adolescents (Chen et al., 2015b). Wertsch (2008) proposes that higher thinking functions develop in the context of interpersonal interaction. Relevant studies have proved that parent–child interaction (Roskam et al., 2014), peer social interaction (Moriguchi et al., 2020), and teacher-student relationship (Suntheimer and Wolf, 2020) have positive effects on executive function and attention control of children and adolescents. Conversely, social anxiety, loneliness and negative emotions can impair executive function in children and adolescents (Frick et al., 2013; Ma et al., 2019). Sports skills, especially open sports skills such as football and basketball, have specific competition rules, and need to cooperate with peers and assume certain role responsibilities. In this interpersonal interaction process, individuals cooperate with the team to complete tasks by inhibiting irrational behavior, which helps to enhance peer relationships and social interactions, and increases the sense of team identity and honor (Gong et al., 2020). In addition, positive exercise experiences and a sense of accomplishment are perceived to increase exercise confidence, enjoyment, and motivation. This leads to a more positive relationship with teachers (Gong et al., 2020), which can facilitate the development of executive functioning.

### Agility, coordination

The above studies show that sequence skills have a greater effect on improving cognitive function in children and adolescents than continuous skills. Sequence skills combine discrete, simple motors into more complex motors, and this complex multi-limb motor characteristic plays a key role in the development of agility and coordination (Zhang, 2012; Diamond, 2015; Chen et al., 2021). Agility and coordination are more complex, integrated motor qualities. Agility refers to the body’s ability to quickly change position, switch movements and improvise during movement (Wang and Su, 2016). Coordination refers to the ability of the body’s organs and systems to cooperate with each other in time and space to complete movements during exercise (Wang and Su, 2016). Neural coordination is the basis of agility and coordination quality. Actions are mainly completed through the synergy and cooperation of the mutual conversion of excitation and inhibition of the neural processes (Young and Willey, 2010). Therefore, the more complex the completed action, the more precise the coordination of the excitation and inhibition process of the cerebral cortex is required (Young and Willey, 2010), and the transformation of this excitation and inhibition reflected in the cognitive explicit behavior is the inhibitory control process. Related studies (Mora-Gonzalez et al., 2019; Xiao et al., 2021; Hu Q. et al., 2022) have also shown a strong link between agility and coordination and the degree of cognitive and brain development in children and adolescents. Open-sequence skills such as ball games, fencing and wrestling with sudden starts, sharp stops and rapid shifts in movement; closed-sequence skills such as aerobics and martial arts routines with complex and varied basic steps and manoeuvres and fast paced movements. These motor skills require the involvement of agility and coordination qualities and help to promote improved cognitive function.

### Cardiorespiratory fitness

Motor skills influence the development of cardiorespiratory fitness by influencing an individual’s choice of physical activity content (Yin, 2019). Motor skills based on aerobic metabolism are more conducive to the development of cardiorespiratory fitness. Some studies (Wang et al., 2001; Yang et al., 2010) have shown that long-distance running is more conducive to improving cardiorespiratory fitness than football, dance, and Tai Chi exercises. There is a clear association between cardiorespiratory fitness and cognition in children and adolescents. Welk et al. (2010) found a significant association between cardiovascular health and academic performance after controlling for potential confounders. Niederer et al. (2011) found an association between cardiorespiratory fitness and better attention in preschoolers, with baseline cardiorespiratory fitness independently associated with improved attention after 9 months of follow-up. In neurobiology, children and adolescents with higher cardiopulmonary fitness have larger hippocampus and basal ganglia, more white matter fiber bundles, and more activation and connection in frontal, temporal, parietal and cerebellar cortex (Erickson et al., 2015; Talukdar et al., 2018). The strength of connectivity in these areas predicts individual executive function, fluid intelligence and academic performance (Erickson et al., 2015; Talukdar et al., 2018).

## Prospects for future study

### Further study on the correlation between motor skills and cognition in children and adolescents

The variety of motor skills and the complexity of the classification system make it impossible to distinguish activity tasks effectively by a single dimension of classification through the predictability of the environmental context or the complexity of the movement structure alone. For example, there are obvious differences in the movement structure between aerobics and middle and long distance running, which belong to the same closed skills. For another example, basketball and Tai Chi, which belong to the same sequence skills, also have obvious differences in environmental context and cognitive participation. Therefore, when exploring the relationship between motor skill type and executive functioning, the interaction between open-closed skills and sequential-continuous skills should be explored in the context of the multidimensional aspects of skill classification. The relationship between motor skill type and cognitive benefits in children and adolescents should be further clarified on the basis of testing evidence from one-dimensional classification studies. Furthermore, the complexity of cognitive processes dictates that the study of motor skills and the cognitive benefits of children and adolescents is a systematic project. However, most of the current studies are fragmented, and lacks systematic integration. The main reason for this is that measures of cognition are often limited by research interests and instruments, and therefore the choice of measures is usually not a complete measure of children’s and adolescents’ cognitive performance. Cognition is a relatively large category, and motor skills are selective for cognitive development (Zhou and Jin, 2021), but which cognitive indicators are more effectively promoted needs to be further explored. Cross-sectional studies are a convenient option for conducting systematic research on the relationship between motor skills and children’s and adolescents’ cognition, but more confounding factors need to be taken into account.

In addition, the relationship between exercise intensity on the treadmill / power bicycle and children’s cognition in the laboratory scenario was “inverted U-shaped” (Chang et al., 2011; Ludyga et al., 2016). However, the relationship between exercise intensity and cognition in the natural environment is moderated by the type of motor skill, so that the relationship between the two does not satisfy the “inverted U-shaped” (Chen et al., 2014). We look forward to further research exploring the interaction between motor skills and exercise intensity on the cognition of children and adolescents. Finally, there are time-course effects of exercise interventions in children’s and adolescents’ cognition. Which exercise time parameters produce the greatest cognitive benefits for different types of skills? How long do the cognitive benefits produced by acute or cyclical interventions for different types of skills last? These questions also need to be answered. Given the fragmentation of research in this area, we suggest that there is a need to clarify the association between types of motor skills and the cognitive development of children and adolescents, and a need to continue to explore the dose-effect relationship between complex motor skill-led physical activity and the cognitive performance of children and adolescents in real-word settings, to clarify the full picture of the relationship between the variables, and to provide a detailed scientific basis for the pedagogical practice of cognitive interventions in school sport.

### Further testing of pathways and mechanisms for motor skills to enhance cognition in children and adolescents

This study proposes pathways and mechanisms by which motor skills enhance the cognitive benefits of children and adolescents (Figure 1), and identifies environmental stimuli, interpersonal interaction, agility, coordination, and cardiorespiratory fitness as cognitive modifiers of motor skills to improve cognition. However, the above pathways and mechanisms are derived through logical reasoning based on an overview of motor skill types and their development in relation to the cognitive benefits of children and adolescents. With the exception of Chen et al. (2015b), who validated that increased interpersonal interaction improves executive function in children and adolescents, the remaining pathways have not been effectively tested. For movement experience, the relationship between motor skill proficiency and cognitive development can be explored by drawing on the Standard Test of Sport Skill Level for Adolescents (STSSLA) (Tang, 2018) and the Game Performance Assessment Instrument (GPAI) (Oslin et al., 1998) to assess the quality of their technical movements and the practical operation of the game situation. For environmental stimuli, experimental studies can be conducted by selecting one motor skill and by manipulating the environmental context (predictable and unpredictable) to test the hypothesis that motor skill teaching practices enhance the cognitive benefits of children and adolescents by increasing the amount of environmental information. For agility, coordination and cardiorespiratory fitness, a cross-sectional study design can be used to examine the mediating role of agility, coordination and cardiorespiratory fitness between motor skill types and cognitive function in children and adolescents through pathway analysis. It is expected that subsequent studies will verify this separately.

### Teaching motor skills in line with the cognitive development of children and adolescents

The essence of education is to use scientific and effective means to promote the cognitive and learning efficiency of the individual based on the laws of brain development. With increasing research in cognitive psychology and cognitive neuroscience, there is a growing focus on the acquisition and restructuring of internal mental representations during motor skill learning (Ertmer and Newby, 1993). “Mind–body monism” also advocates that people are a unity, and the process of motor skills learning is an operational process that combines cognition and practice (Odegard, 1970; Ji et al., 2010). The relationship between motor skills and brain cognition of children and adolescents provides a new perspective for the reform of physical education curriculum. The 2022 Physical Education and Health Standards for Compulsory Education issued by the Chinese Ministry of Education also advocate teaching contextual skills from an individual cognitive perspective, with an emphasis on improving agility and coordination. An overview found that the environmental stimulus information embedded in different types of motor skills, the level of interpersonal interaction required and the degree of effect on agility, coordination and cardiorespiratory fitness were the main factors in improving cognition. Similarly, these factors are also necessary to promote the development of motor skills. Rich environmental stimuli and interpersonal interaction information facilitate cue perception and perceptual decision-making; high levels of agility, coordination and cardiorespiratory fitness facilitate motor execution and motor control. Follow-up studies can incorporate the above factors into motor skills teaching practices to promote the development of cognitive performance and motor skills in children and adolescents. For example, properly improving the cardiorespiratory fitness of children and young people during the teaching of interceptive skills such as table tennis can boost their brain blood oxygen supply and increase the efficiency of brain activation. The teaching of strategic skills such as football emphasizes the role of the environment and people. We can design situational and practical sports games or sports competitions to enhance children’s and adolescents’ group spirit and sports interest, and promote their “awareness” or “ball IQ.” Another example is the inclusion of variable movements such as chasing changes of direction in the teaching of middle distance running skills. This promotes the development of agility and coordination to activate relevant neural pathways and improve brain structure and brain networks. But which skills are more important in teaching practice and which elements of cognitive regulation need to be supplemented? What is the most significant proportion of these elements in different types of skills teaching practice? The above questions also need to be answered through the practice of teaching motor skills, so that the educational function of physical education can be further developed.

## Author contributions

PS designed the research, drafted the article and reviewed relevant literature. XF proofread manuscript and searched relevant literature. All authors participated the intellectual content of the manuscript. All authors contributed to the article and approved the submitted version.

## Funding

The work was supported by the Humanities and Social Sciences youth project of Liaoning provincial department of education (WQ2020012).

## Conflict of interest

The authors declare that the research was conducted in the absence of any commercial or financial relationships that could be construed as a potential conflict of interest.

## Publisher’s note

All claims expressed in this article are solely those of the authors and do not necessarily represent those of their affiliated organizations, or those of the publisher, the editors and the reviewers. Any product that may be evaluated in this article, or claim that may be made by its manufacturer, is not guaranteed or endorsed by the publisher.
