# Supplementary material for: Motor skills and cognitive benefits in children and adolescents: Relationship, mechanism and perspectives
Source: Front Psychol. 2022 Nov 21;13:1017825. doi: 10.3389/fpsyg.2022.1017825 (PMC9721199; doi:10.3389/fpsyg.2022.1017825)
Supplement: Supplementary file 1 [file Table_1.DOCX]

**1 Search strategy**

Relevant literature was searched by one researcher using both English and Chinese search terms. Chinese literature was searched in CNKI, Wan-Fang and VIP core databases; English literature was searched in Web of Science (WOS), PubMed, Scopus and EBSCO databases. The search time frame is from the creation of the database to May 2022. In this study, a combination search was conducted with the following three sets of subject terms: (1) motor skill OR sports items OR sports types OR exercise; (2) executive function OR working memory OR inhibition control OR cognitive flexibility OR self-control OR self-regulation; (3) children OR child OR adolescent OR teenagers OR students. The Boolean logical operator “AND” is used to join three groups of subject terms.

**2** **Selection criteria**

Inclusion and exclusion criteria for the literature were designed according to the PICOS principles. Inclusion criteria were as follows: (1) subjects were typical and atypical children and adolescents; (2) interventions are acute and long-term exercise interventions in real-word settings; (3) control measures include traditional physical education courses, basic academic courses, free activities or sitting; (4) outcome variables include planning, inhibitory control, working memory, cognitive flexibility; (5) study designs include randomized controlled trial (RCT), randomized crossover design (RCD), non-randomized concurrent control trial (non-RCCT) and before-after study (BAS). Exclusion criteria were as follows: (1) cross-sectional, case-control, historical data review and other descriptive studies; (2) overviews, abstracts, letters, reviews, etc; (3) screen-based physical games, such as Xbox and Kinect; (4) combined interventions of physical exercise and cognitive therapy; (5) for duplicate publications, only the higher quality literature was included. The literature selection process follows the order of title, abstract, figure, and full text. Literature screening was carried out independently by two researchers each, with two other researchers conducting a secondary assessment of the screened literature. If there was a dispute between the two groups, all researchers discussed and agreed together.

**3 Data extraction**

Extracted data including first author, year of publication, study design, subject characteristics, interventions, control measures and outcome variables were entered into Excel 2010 and saved. The data extraction was carried out independently by two researchers and the extracted data was assessed secondarily by two other researchers. If there was a dispute between the two groups, all researchers discussed and agreed together.

**4 Results**

**3.1 Literature selection results**

The search strategy retrieved a total yield of 7240 articles. The retrieved articles were imported into EndNote X9 software for de duplication, and 2311 articles were obtained. After further screening of articles, a total of 65 articles were finally included. Finally, the study included a comparative study of different types of motor skill interventions, as shown in Table 1 and Table 2 of our paper.

**Table1. Characteristics of included studies**

| Included articles  Study design | Patients（N/Age/F%） | Interventions and controls | Outcome | |
| --- | --- | --- | --- | --- |
|  |  |  | Outcome measures | Results |
| **Acute interventions for typical children and adolescents** | | | | |
| Niemann et al., 2013  RCT | E=27/9.7±0.4y/NC  C=15/9.7±0.5y/NC | 12min high intensity (85-90% HRmax) track and field run (Cl, Cn) (E) vs. sitting (C) | ②d2-test | + |
| Palmer et al., 2013  RCD | 16/49.4±5.3m/18.8% | 30min of passing, dribbling and throwing activities (O, S) (E) vs. sitting (C) | ②PDTP | 0 |
| Yan et al., 2014  RCT | E1=52/9.8±0.3y/53.8%  E2=51/9.7±0.3y/49.0%  C=51/9.8±0.3y/49.0% | 30min moderate intensity (60-69% HRmax) aerobics (Cl, S) (E1) vs. obstacle run (O, Cn) (E2) vs. sitting (C) | ②Flanker (E1＞E2)  ③1-back (E1＞E2)  ④More-odd shifting (E2＞E1) | +&  +&  +& |
| Chen et al., 2014a  RCT | E1=30/9.8±0.3y/50.0%  E2=30/9.8±0.3y/53.3%  E3=32/9.7±0.3y/46.9%  C=28/9.8±0.3y/50.0% | 30min low intensity (50-59% HRmax) basketball high dribbling and dribbling between runs (Cl, S) (E1) vs. moderate intensity (60-69% HRmax) (E2) vs. high intensity (70-79% HRmax) (E3) vs. free activities in their classroom (C) | ②Flanker (E2＞E1=E3＞C)  ③1-back (E2=E3＞E1=C)  ④More-odd shifting (E2＞E3=C＞E1) | +&  +0&  +­0& |
| Chen et al., 2014b  RCT | E=44/3~5g/47.7%  C=38/3~5g/55.3% | 30min moderate intensity (60-70% HRmax) track and field run (Cl, Cn) (E) vs. sedentary reading (C) | ②Flanker  ③2-back  ④More-odd shifting | +  +  + |
| Chen et al., 2015a  RCT | E1=39/9.1±0.3y/48.7%  E2=38/9.1±0.3y/44.7%  C=38/9.2±0.4y/77.7% | 30min moderate intensity (60-69% HRmax) cooperative rope skipping (O, Cn) (E1) vs. single rope skipping (Cl, Cn) (E2) vs. sedentary reading (C) | ②Flanker (E1＞E2)  ③1-back (E1＞E2)  ④More-odd shifting (E1＞E2) | +&  +&  +& |
| Chen et al., 2015b  RCT | E=24/9.5±0.3y/NC  C=22/9.5±0.3y/NC | 30min moderate intensity (60-69% HRmax) basketball high dribbling and dribbling between runs (Cl, S) (E) vs. free activities in their classroom (C) | ②Flanker (E1＞C)  ③1-back (E1＞C)  ④More-odd shifting (E1＞C) | +  +  + |
| Jäger et al, 2015  RCT | E1=54/134.6±6.6m/64.8%  E2=62/135.3±6.5m/45.2%  E3=60/136.3±6.8m/50.0%  C=58/135.8±6.3m/56.9% | 20min moderate intensity (70% HRmax) cognitive involvement skill games (O,S) (E1) vs. aerobic exercise without cognitive involvement (Cl, Cn) (E2) vs. meditation with cognitive involvement (E3) vs. sitting without cognitive involvement (C) | ②Flanker (E1=E2=E3)  ③1-back (E1=E2=E3）  ④More-odd shifting (E1=E2=E3) | 0&  0&  0& |
| Gallotta et al., 2015a  RCT | E1=31/8~11y/NC  E2=46/8~11y/NC  C=39/8~11y/NC | 50min traditional PE course (brisk walking, jogging, jumping, etc.) (Cl, Cn) (E1) vs. basketball skills acquisition practice (O,S) (E2) vs. basic academic course (C) | ②d2-test (E1＞C＞E2) | +-& |
| Cooper et al., 2016  RCD | 44/12.6±0.6/52.3% | 10min high intensity interval sprint in athletics hall (Cl, Cn) (E) vs. sitting (C) | ②Stroop  ③Corsi blocks test  ④DSST | +  0  0 |
| Stein et al., 2017  RCT | E=48/72.2±5.2m/50.0%  C=53/72.3±6.9m/52.8% | 20min motor skill learning practice based on coordination of both sides of the body (Cl, S) (E) vs. board game (C) | ②Simon-says task  ②Hearts and Flowers task-incongruent block  ④Hearts and Flowers task-mixed block | +  0  0 |
| O’Brien et al., 2021  RCT | E1=16/7.0±0.5y/0.0%  E2=16/6.7±0.1y/0.0%  C=19/7.0±0.5y/0.0% | 30min open skills activities such as basketball, football, tennis (O,S) (E1) vs. closed skills activities such as race, rope skipping, circuit training (Cl,Cn) (E2) vs. free activities in their classroom (C) | ③Backward Digit Span (E1＞E2)  ③Corsi blocks test  ③Motor span task (E2＞E1) | +&  0  +& |
| Ottoboni et al., 2021  RCT | 125/7~10y/NC | 30min high intensity (170~180 bpm) team ball games (O,S) (E1) vs. agility obstacle run (O,Cn) (E2) vs. basic academic course (C) | ③Digit Span (E1＞E2)  ③Corsi blocks test (E1＞E2) | +&  +& |
| **Long-term interventions for typical children and adolescents** | | | | |
| Manjunath et al., 2001  RCT | E=10/10~13y/100.0%  C=10/10~13y/100.0% | 4 weeks (7 x/week) yoga intervention (Cl, S), 75min/time (E) vs. traditional PE course (C) | ①Tower of London | + |
| Lakes et al., 2004  RCT | 207/Kindergarten to Primary 5/NC | 12 weeks (2-3 x/week) martial arts intervention (Cl, S), 45min/time (E) vs. traditional PE course (C) | ③Digit Span | 0 |
| Davis et al., 2011  RCT | E1=55/7~11y/NC  E2=56/7~11y/NC  C=60/7~11y/NC | 13 weeks (7x/week) moderate intensity (>150 bpm) running games, rope skipping, football and basketball exercise intervention (combined skills), 20min/time (E1) vs. 40min/time (E2) vs. blank control (C) | ①Cognitive Assessment System-Planning (E2＞E1=C) | +0& |
| Kamijo et al., 2011  RCT | E=20/8.9±0.5y/55.0%  C=16/9.1±0.6y/50.0% | 36 weeks (7x/week) moderate to high intensity physical training combined with dribbling skills practice (combined skills), 70 min/time (E) vs. blank control (C) | ③Sternberg | + |
| Chang et al., 2013  non-RCCT | E1=13/7.2±0.3/46.2%  E2=13/7.0±0.3/53.9% | 8 weeks (2w/week) low intensity (40-50% HRmax) football learning practice (O, S), 2 sessions/week, 35min/time (E1) vs. moderate intensity (60-70% HRmax) (E2) vs. pre-test | ②Flanker  (E1：Post＞Pre；E2：Post＞Pre) | + |
| Lakes et al., 2013  RCT | E=50/12.2y/52.00%  C=31/12.3y/48.00% | 36 weeks (2x/week) Taekwondo (Cl, S), 45min/time (E) vs. traditional PE course (C) | ②Parental rated inhibitory control  attention control  behavior control  ②Hearts and Flowers task-incongruent block  ④Hearts and Flowers task-mixed block | 0  +  0  0 |
| Telles et al., 2013  RCT | E1=49/10.4±1.2y/30.6%  E2=49/10.5±1.3y/46.9% | 12 weeks (5x/week) yoga (Cl, S), 45min/time (E1) vs. physical exercise such as jogging, sprint running, relay races (Cl, Cn) (E2) vs. pre-test | ②Stroop (E1＜E2) | +& |
| Crova et al., 2014  RCT | E=37/9.6±0.5y/46.0%  C=33/9.6±0.5y/54.6% | 21 weeks (1x/week) moderate intensity (150.5±6.4 bpm) tennis (O, S), 120min/ time (E) vs. traditional PE course (C) | ②RNG-inhibition of mental routines  ③RNG-working memory updating | +  0 |
| Yin et al., 2014  RCT | 326/3~5g/47.9% | 20 weeks (3x/week) moderate intensity (120-140 bpm) martial arts + rope skipping+ 8 word run (Cl), 30min/time (E1) vs. pattern running (O, Cn), 5x/week (E2) vs. blank control (C) | ②Flanker (E1＞E2=C)  ③2-back (E2＞E1)  ④More-odd shifting (E1=E2) | +0&  +&  +& |
| Krafft et al., 2014a  RCT | E=10/9.9±0.6y/50.0%  C=8/9.4±0.8y/50.0% | 32 weeks (7x/week) aerobics (Cl, S), 40min/time (E) vs. sedentary attention control (C) | ⑤BRIEF | + |
| Krafft et al., 2014b  RCT | E=24/9.7±0.8y/71%  C=19/9.9±0.9y/58% | 32 weeks (7x/week) moderate intensity (161±9 bpm) rope skipping and tag games (combined skills) 40min/time (E) vs. sedentary attention control (C) | ②Flanker  ②Antisaccade task | +  + |
| Yin et al., 2015  RCT | 610/3~5g/46.9% | 10 weeks (5week) 40-80% HRmax pattern running (O, Cn), 30min/time (E1) vs. fun track and field games (O, Cn), 3x/week (E2) vs. small handball and physical fitness exercises (combined skills), 3x/week (E3) vs. shuttlecock and games (combined skills) (E4) vs. martial arts, rope skipping, 8 word run (Cn), 3x/week (E5) vs. regular extra-curricular physical activity (C) | ②Flanker  (E5＞E4＞E2＞E1=E3=C)  ③2-back  (E1=E5＞E2=E3=E4=C)  ④More-odd shifting  (E1=E5＞E3＞E2=E4=C) | +0&  +0&  +0& |
| Jiang et al., 2015  RCT | E=31/5~6y/NC  C=30/5~6y/NC | 8 weeks (2x/week) moderate intensity (60-70% HRmax) football games (O, S), 35 min/time (E) vs. blank control (C) | ②Panda-Lion task  ②Snow-Grass task  ③Corsi blocks test  ③Reverse Corsi blocks test  ④Flexible Item Selection task | +  +  0  0  0 |
| Schmidt et al., 2015  RCT | E1=69/11.3±0.6y/62.3%  E2=57/11.3±0.6y/50.9%  C=55/11.4±0.6y/49.1% | 6 weeks (2x/week) high intensity soft hockey and basketball games (O, S), 45min/time (E1) vs. 200m round trip run (Cl, Cn) (E2) vs. traditional PE course (C) | ②Flanker (E1＞E2=C)  ③2-back (E1＞E2=C)  ④More-odd shifting (E1＞E2=C) | +0&  +0&  +0& |
| Gallotta et al., 2015b  RCT | E1=56/8~11y/NC  E2=59/8~11y/NC  C=41/8~11y/NC | 20 weeks (2x/week) moderate intensity (RPE=5-8) traditional PE course focusing on cardiovascular fitness, 60 min/time (E1) vs. activities focusing on improving coordination and flexibility (E2) vs. blank control (C) | ②d2-test | +0& |
| Chen et al., 2016a  RCT | E=20/11.4±0.6y/NC  C=20/11.3±06y/NC | 8 weeks (3x/week) moderate intensity (60-69% HRmax) mind-body aerobics (Cl, S), 40min/time (E) vs. regular academic (C) | ②Flanker  ③1-back  ④More-odd shifting | 0  +  + |
| Koutsandreou et al., 2016  RCT | E1=27/9.3±0.6y/NC  E2=23/9.4±0.7y/NC  C=21/9.3±0.6y/NC | 10 weeks (3x/week) moderate intensity (60-70% HRmax) aerobic exercise, 45min/time (E1) vs. moderate intensity (55%-65% HRmax) skill practice focused on improving coordination (S) (E2) vs. supported work (C) vs.do their homework（C） | ③Letter Digit Span (E2＞E1) | +& |
| Alesi et al., 2016  non-RCCT | E=24/8.8±1.1y/0.0%  C=20/9.3±0.9y/0.0% | 24 weeks (2x/week) football intervention (O, S), 75min/time (E) vs. traditional PE course, 1 x/week, 60min/time (C) | ①Tower of London  ③Forward Digit Span  ③Backward Digit Span  ③Corsi blocks test | +  0  0  + |
| Pesce et al., 2016  RCT | E=232/5~10y/50.4%  C=228/5~10y/49.6% | 24 weeks (1x/week) moderate intensity (131.9±17.4 bpm) skill games (O, S) focusing on motor coordination and cognitive engagement, 60min/time (E) vs. traditional PE course (C) | ②RNG-inhibition of mental routines  ③RNG-working memory updating | +  0 |
| Robinson et al., 2016  RCT | E=68/52.4±5.2m/39.7%  C=45/51.6±5.2m/40.0% | 5 weeks (3x/week) Children’s Health Activity Programme (motor skills-led intervention), 40min/time (E) vs. outdoor free play (C) | ②Delay of gratification snack task | + |
| van der Niet et al., 2016  non-RCCT | E=53/8.8±0.8y/64.2%  C=52/8.9±1.2y/38.5% | 22 weeks (2x/week) of moderate to high intensity running games, circuit training and football with cognitive participation (combined skills), 30min/time (E) vs. blank control (C) | ①Tower of London  ②Stroop  ③Visual Memory Span  ③Digit Span  ④Trailmaking test | 0  +  0  +  0 |
| Chen et al., 2017  RCT | E=21/9.4±0.5y/47.6%  C=20/9.2±0.4y/50.0% | 8 weeks (2x/week) moderate intensity (60-69% HRmax) football intervention (O, S), 40min/time (E) vs. traditional PE course (C) | ②Flanker  ③1-back  ④More-odd shifting | +  +  + |
| Cho et al., 2017  RCT | E=15/11.2±0.8y/40.0%  C=15/11.3±0.7y/40.0% | 16 weeks (5x/week) moderate intensity (RPE=11-15) taekwondo intervention (O, S), 60 min/time (E) vs. blank control (C) | ②Stroop | + |
| Xiong et al., 2017  non-RCCT | 39/4.67y/48.7% | 12 weeks (7x/week) structured motor skills intervention (combined skills), 30min/time (E) vs. unstructured free play (C) | ④WCST | + |
| Hsieh et al., 2017  non-RCCT | E=24/8.7±1.1y/NC  C=20/8.6±1.1y/NC | 8 weeks (2x/week) moderate intensity (136.4±16.8 bpm) gymnastic intervention (Cl, S), 90 min/time (E) vs. blank control (C) | ③Delayed matching-to-sample test | + |
| Mulvey et al., 2018  RCT | T=50/3~6y/NC  C=57/3~6y/NC | 6 weeks (2x/week) SKIP program, 30min/time (E) vs. rest as usual (C) | ②HTKS | + |
| Dai et al., 2020  non-RCCT | E=46/10.5±0.3y/NC  C=43/10.4±0.3y/NC | 24 weeks (5x/week) moderate intensity (60~69% HRmax) football intervention (O, S), 120min/time (E) vs. blank control (C) | ②Flanker  ③2-back  ④Salthouse | +  +  + |
| Lai et al., 2020  RCT | E=10/5~7y/50.0%  C=10/5~7y/50.0% | 8 weeks (2x/week) moderate intensity (60~69% HRmax) tennis intervention (O, S), 60min/time (E) vs. basic academic course (C) | ③1-back | + |
| Oppici et al., 2020  RCT | E1=30/8.8±0.5y/62.0%  E2=30/8.7±0.7y/59.0%  C=20/8.9±0.7y/63.0% | 7 weeks (2x/week) high cognitive dance practice (Cl, S), 60min/time (E1) vs. low cognitive dance practice (Cl, S) (E2) vs. blank control (C) | ②Flanker  ③List Sorting Working Memory test  ④Dimensional Change Card Sort test | 0  0  0 |
| Ma et al., 2022  non-RCCT | E=40/9.2±0.3y/NC  C=40/9.2±0.3y/NC | 16 weeks (3x/week) football intervention (O, S), 40min/time (E) vs. blank control (C) | ②GO/NO GO  ③1-back  ③2-back  ④More-odd shifting | +  +  +  + |
| **Long-term interventions for atypical children and adolescents** | | | | |
| Tsai et al., 2009  RCT | DCD  E=14/9~13y/NC  C=13/9~13y/NC | 10 weeks (3x/week) table tennis intervention (O, S), 50 min/time (E) vs. blank control (C) | ②Endogenous Posner task | + |
| Verret et al., 2012  non-RCCT | ADHD  E=10/9.1±1.1y/NC  C=11/9.1±1.1y/NC | 10 weeks (3x/week) progressive aerobic exercise, muscle training and ball activities such as basketball and football (combined skills), 45min/time (E) vs. blank control (C) | ②walk/don’t walk | 0 |
| Smith et al., 2013  BAS | ADHD  14/6.7±1.0y/NC | 9 weeks (7x/week) moderate to high displacement skills such as jumping, running, crab walking (Cl, Cn), 30min/time (E) vs. pre-test | ①Mazes  ②Simon Says  ②Red Light/Green Light  ③Finger Windows  ③Sentence Memory | 0  0  +  0  0 |
| Westendorp et al., 2014  RCT | LD  E=43/9.4±0.83y/NC  C=44/9.1±0.96y/NC | 16 weeks (2x/week) ball skills with higher cognitive requirements (O, S), 240min/time (E) vs. blank control (C) | ①Tower of London  ④Trail Making Test | 0  0 |
| Chen et al., 2015c  RCT | deafness  E=18/11.7±1.2y/27.8%  C=15/11.9±1.5y/33.3% | 8 weeks (3x/week) moderate intensity (60-69% HRmax) fancy rope skipping (Cl, S), 40min/time (E) vs. daily activity (C) | ②Flanker  ③1-back  ④More-odd shifting | 0  +  + |
| Ziereis et al., 2015  RCT | ADHD  E1=13/9.2±1.3y/NC  E2=14/9.6±1.6y/NC  C=16/9.5±1.4y/NC | 12 weeks (1x/week) to improve skills in ball control, balance and manual dexterity (O, S), 60min/time (E1) vs. exercise without specific focus (E2) vs. blank control (C) | ③Forwards/Backwards Digit Span (E1=E2)  ③Letter-Number-Sequencing task (E1=E2) | +&  +& |
| Choi et al., 2015  RCT | ADHD  E=13/15.8±1.7y/NC  C=17/16.0±1.2y/NC | 6 weeks (3x/week) moderate intensity (60% HRmax) shuttle run, furlong run, rope skipping and basketball (combination skills), 90min/time (E) vs. behavioural control education course (C) | ④WCST | + |
| Chen et al., 2015  RCT | MR  E1=46/10.9±3.9y/43.5%  E2=45/10.6±3.6y/46.7%  C=41/10.7±4.0y/43.9% | 16 weeks (3x/week) sensory integration, neurodevelopmental therapy and perceptual-motor therapy, 60 min/time (E1) vs. table tennis (O, S) (E2) vs. blank control (C) | ②Stroop (E2＞E1)  ④WCST (E2＞E1) | +&  +& |
| Chen et al., 2016b  RCT | deafness  E=14/11.4±0.5y/41.7%  C=12/11.0±0.6y/42.9% | 11 weeks (4x/week) moderate intensity (60-69% HRmax) 8-figure running, martial arts exercises and fancy rope skipping (combination skills), 30min/time (E) vs. regular activity (C) | ③2-back | + |
| Pan et al, 2016a  RCT | 1=LD; 2=Normal  E1=23/12.0±0.6y/NC  E2=25/12.0±0.6y/NC  C1=22/12.0±0.6y/NC  C2=23/12.0±0.6y/NC | 10 weeks (3x/week) moderate intensity (60-69% HRmax) basketball intervention (O, S), 30 min/time (E) vs. traditional PE course (C) | ②Flanker (E1＞E2)  ③1-back (E1＞E2)  ④More-odd shifting (E1＞E2) | +&  +&  +& |
| Pan et al., 2016b  non-RCCT | LD  E=36/6g/NC  C=23/6g/NC | 10 weeks (3x/week) moderate intensity (60-69% HRmax) martial arts exercises, fancy shuttlecock, cooperative games (combination skills), 30min/time (E) vs. blank control (C) | ②Flanker  ③1-back  ④More-odd shifting | +  0  0 |
| Pan et al., 2016  RCT | ADHD  E=20/8.9±1.5y/NC  C=20/8.9±1.6y/NC | 12 weeks (2x/week) table tennis intervention (O, S), 70 min/time (E) vs. regular activity (C) | ②Stroop | + |
| Pan et al., 2017  RCT | 1=LD，2=Normal  E1=26/4g/NC  E2=26/4g/NC  C1=23/4g/NC  C2=21/4g/NC | 16 weeks (3x/week) moderate intensity (60-69% HRmax) basketball intervention (O, S), 30 min/time (E) vs. blank control (C) | ②Flanker (E1＞E2)  ③1-back (E1=E2)  ④More-odd shifting (E1＞E2) | +&  +&  +& |
| Liu et al., 2018  RCT | ADHD  E=32/NC/50.0%  C=32/NC/50.0% | 14 weeks (3x/week) moderate intensity (60-70% HRmax) orienteering (O, Cn), 35min/time (E) vs. traditional PE course (C) | ③Corsi blocks test | + |
| Chen et al., 2018  RCT | deafness  E=8/11.8±1.0y/50.0%  C=8/11.3±1.2y/37.5% | 11 weeks (4x/week) moderate intensity (60-69% HRmax) fancy rope skipping, martial arts exercises and fancy running (combination skills), 45min/time (E) vs. regular activity (C) | ②Flanker | + |
| Yin et al., 2018  RCT | 1a= Chinese LD,  1b= mathematical LD,  1c= both LD,  2a,2b,2c = Normal  E1a=24/NC/NC  E1b=25/NC/NC  E1c=23/NC/NC  E2a=23/NC/NC  E2b=23/NC/NC  E2c=25/NC/NC  C1a=20/NC/NC  C1b=22/NC/NC  C1c=22/NC/NC  C2a=25/NC/NC  C2b=23/NC/NC  C2c=23/NC/NC | 10 weeks (3x/week) moderate intensity (60-69% HRmax) fun games, fancy rope skipping and fancy running (combination skills), 30min/time (E1a, E2a) vs. cooperative games, martial arts exercises, fancy shuttlecock (combination skills) (E1b, E2b) vs. basketball (O, S) (E1c, E2c) vs. traditional PE course (C) | ②Flanker  （E1a=C1a; E2a=C2a; E1a=T2a; E1b＞C1b; E2b＞C2b; E1b＞E2b; E1c＞C1c; E2c＞C2c; E1c＞E2c）  ③1-back  （E1a＞C1a; E2a＞C2a; E1a＞E2a; E1b=C1b; E2b=C2b; E1b=E2b; E1c＞C1c; E2c＞C2c; E1c＞E2c）  ④More-odd shifting  （E1a=C1a; E2a=C2a; E1a=E2a; E1b=C1b; E2b=C2b; E1b=E2b; E1c＞C1c; E2c＞C2c; E1c＞E2c） | +0&  +0&  +0& |
| Kadri et al., 2019  RCT | ADHD  E=20/14.5±3.5y/10.0%  C=20/14.2±3.0y/10.0% | 72 weeks (2x/week) Taekwondo (O, S), 50min/time (E) vs. traditional PE course (C) | ②Stroop | + |
| Pan et al., 2019  non-RCCT | ADHD  E=15/9.1±1.4y/NC  C=15/8.9±1.7y/NC | 12 weeks (2x/week) table tennis intervention (O, S), 70 min/time (E) vs. regular activity (C) | ②Stroop  ④WCST | +  - |
| Song et al., 2020  RCT | 1=ADHD，2=Normal  E1=22/NC/45.5%  E2=20/NC/50.0%  C1=22/NC/45.5%  C2=20/NC/50.0% | 10 weeks moderate intensity (60-69% HRmax) orienteering (O, Cn), 25-35min/time (E) vs. traditional PE course (C) | ②Flanker (E1＞E2)  ③1-back (E1＞E2)  ④More-odd shifting (E1＞E2) | +&  +&  +& |
| Silva et al., 2020  RCT | ADHD  E=18/12.0±1.0y/NC  C=15/12.0±2.0y/NC | 8 weeks (2x/week) swimming intervention (Cl, Cn), 45 min/time (E) vs. blank control (C) | ④Cancellation Attention Test | + |
| Tse et al., 2021  RCT | ASD  E1=22/10.2±0.7y/13.6%  E2=20/9.6±1.6y/20.0%  C=20/9.9±1.3y/25.0% | 2 weeks (5x/week) low intensity (RPE=3-5) learning to cycle in a natural environment (O, Cn), 60min/time (E1) vs. ride a stationary bike (Cl, Cn) (E2) vs. daily activity (C) | ①Tower of London (E1＞E2=C)  ②GO/NO GO(E1＞E2=C)  ③Corsi blocks test(E1＞E2=C)  ③Forwards/Backwards Digit Span (E1=E2=C/E1=C＞E2) | +0&  +0&  +0&  -0& |
| Liang et al., 2022  RCT | ADHD  E=40/8.4±1.4y/25.0%  C=40/8.3±1.3y/20.0% | 12 weeks (3x/week) moderate to high intensity (60-80% HRmax) rope skipping, rope ladder, taekwondo, basketball, table tennis and badminton (combination skills), 60min/time (E) vs. daily activity (C) | ①Tower of London  ②Flanker  ④Cancellation Attention Test | +  +  + |
| Abbreviations and notes: RCT: randomized controlled trial; RCD: randomized crossover design; non-RCCT: non-randomized concurrent control trial; BAS: before-after study; E: Experimental group; C: Control group; y: year; m: month; g: grade; F%: Females as a percentage of subjects; O: Open skills; Cl: Closed skills; Co: Contionous skills; S: Sequence skills; NC: Not clear; HRmax: Maximum heart rate; RPE: Rating Of Perceived Exertion;①: Planning; ②; Inhibitory control; ③: Working memory; ④: Cognitive flexibility; ⑤: Behavioural assessment of executive functions; +: Beneficial to experimental group; -: Beneficial to control group; 0: No significant difference between the experimental and control groups; &: Comparison of intervention results between experimental groups; Pre: Pre-test; Post: Post-test; PDTP: Picture Deletion Task for Preschoolers; DSST: Digit Symbol Substitution Test; RNG: Random Number Generation task; BRIEF: Behavioral Rating Inventory of Executive Function; WCST: Wsiconsin Card Sorting Test; HTKS: Head Toe Knee Shoulder test; DCD: Developmental Coordination Disorder; ADHD: Attention Deficit Hyperactivity Disorder; ASD: Autism Spectrum Disorder; LD: Learning Difficulties; MR: Mental Retardation. | | | | |

Niemann C,Wegner M,Voelcker-Rehage C,et al.Influence of acute and chronic physical activity on cognitive performance and saliva testosterone in preadolescent school children [J].Mental Health and Physical Activity,2013,6(3):197-204.

Palmer K K,Miller M W,Robinson L E.Acute exercise enhances preschoolers’ ability to sustain attention[J].J Sport Exerc Psychol,2013,35(4):433-437.

Yan J,Wang Y,Chen A G,et al.Empirical study of the impact of various school-term physical activity of moderate intensity on the executive function of children in their preadolescence[J].Journal of Sports and Science,2014,35(6):94-100.

Chen A G,Zhao L,Li H Y,et al.Effects of acute basketball dribbling training of different intensity on executive function of primary students[J].Journal of TUS,2014,29(4):352-355.

Chen A G,Yan J,Yin H C,et al.Effects of acute aerobic exercise on multiple aspects of executive function in preadolescent children[J].Psychology of Sport and Exercise,2014,15 (6):627-636.

Chen A G,Zhao Z Y,Yan J.Effects of rope skipping with different forms of organization on the executive function of preadolescent children:a school-based experimental study[J]. Chin J Sports Med,2015,34(9):886-890.

Chen A G,Feng L,Zhu L N,et al.Effect of moderate intensity basketball dribbling teaining of different durations on children’ executive function[J].Journal of Capital University of Physical Education and Sports,2015,27(3):223-227.

Jäger K,Schmidt M,Conzelmann A,et al.The effects of qualitatively different acute physical activity interventions in real-world settings on executive functions in preadolescent children [J].Mental Health and Physical Activity, 2015,9(8):1-9.

Gallotta M C,Emerenziani G P,Franciosi E,et al.Acute physical activity and delayed attention in primary school students[J].Scandinavian journal of medicine & science in sports,2015,25 (3):331-338.

Cooper S B,Bandelow S,Nute M L,et al.Sprint-based exercise and cognitive function in adolescents[J].Preventive medicine reports,2016,4(4):155-161.

Stein M,Auerswald M,Ebersbach M.Relationships between motor and executive functions and the effect of an acute coordinative intervention on executive functions in kindergartners[J].Frontiers in psychology,2017,8(5):859-872.

O’Brien J,Ottoboni G,Tessari A,et al.Multisensory perception,verbal,visuo-spatial and motor working memory modulation after a single open-or closed-skill exercise session in children [J].Journal of Cognitive Enhancement,2021,5(2):141-154.

Ottoboni G,Ceciliani A,Tessari A.The effect of structured exercise on short-term memory subsystems:new insight on training activities[J].International Journal of Environmental Research and Public Health,2021,18(14):7545-7555.

Manjunath N K,Telles S.Improved performance in the Tower of London test following yoga [J].Indian journal of physiology and pharmacology,2001,45(3):351-354.

Lakes K D,Hoyt W T.Promoting self-regulation through school-based martial arts training [J].Journal of Applied Developmental Psychology,2004,25(3):283-302.

Davis C L,Tomporowski P D,McDowell J E,et al.Exercise improves executive function and achievement and alters brain activation in overweight children:a randomized, controlled trial [J].Health psychology,2011,30(1):91-97.

Kamijo K,Pontifex M B,O’Leary K C,et al.The effects of an afterschool physical activity program on working memory in preadolescent children[J].Developmental science,2011,14 (5):1046-1058.

Chang Y K,Tsai Y J,Chen T T,et al.The impacts of coordinative exercise on executive function in kindergarten children:an ERP study[J].Experimental Brain Research,2013,225 (2):187-196.

Lakes K D,Bryars T,Sirisinahal S,et al.The healthy for life taekwondo pilot study:a preliminary evaluation of effects on executive function and BMI,feasibility,and acceptability[J].Mental health and physical activity,2013,6(3):181-188.

Telles S,Singh N,Bhardwaj A K,et al.Effect of yoga or physical exercise on physical, cognitive and emotional measures in children:a randomized controlled trial[J].Child and adolescent psychiatry and mental health,2013,7(1):1-16.

Crova C,Struzzolino I,Marchetti R,et al.Cognitively challenging physical activity benefits executive function in overweight children[J].Journal of sports sciences,2014,32(3): 201-211.

Yin H C,Chen A G,Ma Z,et al.A fllow-up study on two kinds of exercise intervention programs for children’s executive functions[J].China Sport Science,2014,34(3):24-28.

Krafft C E,Schaeffer D J,Schwarz N F,et al.Improved frontoparietal white matter integrity in overweight children is associated with attendance at an after-school exercise program[J]. Developmental Neuroscience,2014,36(1):1-9.

Krafft C E,Schwarz N F,Chi L,et al.An 8-month randomized controlled exercise trial alters brain activation during cognitive tasks in overweight children[J].Obesity,2014,22(1): 232-242.

Yin H C,Li X N,Chen A G,et al.Experimental study on the effects of five kinds of exercise intervention programs on brain executive functions of primary students[J].Journal of TUS,2015,30(1):7-10.

Jiang D L,Zeng C Z.The effect of 8-week soccer exercise with moderate intensity on executive function in preschool children[J].China Sport Science and Technology,2015,51 (2):43-50.

Schmidt M,Jäger K,Egger F,et al.Cognitively engaging chronic physical activity,but not aerobic exercise,affects executive functions in primary school children:a group randomized controlled trial[J].Journal of Sport and Exercise Psychology,2015,37(6):575-591.

Gallotta M C,Emerenziani G P,Iazzoni S,et al.Impacts of coordinative training on normal weight and overweight/obese children’s attentional performance[J].Frontiers in Human Neuroscience,2015,9(12):577-595.

Chen A G,Liang H Y,Yan J,et al.The developmental features of executive functions of left-at-home children and the interventions by mind-body exercise[J].Chinese Journal of Special Education,2016,32(11):69-74.

Koutsandreou F,Wegner M,Niemann C,et al.Effects of motor vs. cardiovascular exercise training on children’s working memory[J].Medicine and Science in Sports and Exercise, 2016,48(6):1144-1152.

Alesi M,Bianco A,Luppina G,et al.Improving children's coordinative skills and executive functions:the effects of a football exercise program[J].Perceptual and motor skills,2016, 122(1):27-46.

Pesce C,Masci I,Marchetti R,et al.Deliberate play and preparation jointly benefit motor and cognitive development:mediated and moderated effects[J].Frontiers in psychology, 2016,7(3): 349-356.

Robinson L E,Palmer K K,Bub K L.Effect of the children’s health activity motor program on motor skills and self-regulation in head start preschoolers:an efficacy trial[J].Frontiers in public health,2016,4(2):173-181.

van der Niet A G,Smith J,Oosterlaan J,et al.Effects of a cognitively demanding aerobic intervention during recess on children’s physical fitness and executive functioning[J]. Pediatric exercise science,2016,28(1):64-70.

[64] Chen A G,Chen L P,Yan J.Exepeimental study on the effect of eight-week football program on executive function among left-behind children[J].Journal of Shandong Sport University, 2017,33(1):85-89.

Cho S Y,So W Y,Roh H T.The effects of taekwondo training on peripheral neuroplasticity- related growth factors,cerebral blood flow velocity,and cognitive functions in healthy children:a randomized controlled trial[J].International journal of environmental research and public health,2017,14(5):454-463.

Xiong S,Li X,Tao K.Effects of structured physical activity program on Chinese young children’s executive functions and perceived physical competence in a day care center[J]. BioMed research international,2017,23(8):1-7.

Hsieh S S,Lin C C,Chang Y K,et al.Effects of childhood gymnastics program on spatial working memory[J].Medicine and science in sports and exercise,2017,49(12):2537-2547.

Mulvey K L,Taunton S,Pennell A,et al.Head,toes,knees,SKIP! Improving preschool children’s executive function through a motor competence intervention[J].Journal of Sport and Exercise Psychology,2018,40(5):233-239.

Dai C.Effects of soccer exercise and stop practice on executive function of school age children[J].Journal of Chengdu Sport University,2020, 46(5):109-113.

Lai Y,Wang Z,Yue G H,et al.Determining whether tennis benefits the updating function in young children:a functional near-Infrared spectroscopy study[J].Applied Sciences,2020,10 (1):407-421.

Oppici L,Rudd J R,Buszard T,et al.Efficacy of a 7-week dance (RCT) PE curriculum with different teaching pedagogies and levels of cognitive challenge to improve working memory capacity and motor competence in 8-10 years old children[J].Psychology of Sport and Exercise,2020,50(2):101675-101723.

Ma Y D,Xu L,Fu Q.An empirical study of the impact of campus football training on children’s mental health[J].Journal of Shenyang Sport University,2022,41(1):58-66.

Tsai C L.The effectiveness of exercise intervention on inhibitory control in children with developmental coordination disorder:using a visuospatial attention paradigm as a model[J]. Research in developmental disabilities,2009,30(6):1268-1280.

Verret C,Guay M C,Berthiaume C,et al.A physical activity program improves behavior and cognitive functions in children with ADHD:an exploratory study[J].Journal of attention disorders,2012,16(1):71-80.

Smith A L,Hoza B,Linnea K,et al.Pilot physical activity intervention reduces severity of ADHD symptoms in young children[J].Journal of attention disorders,2013,17(1):70-82.

Westendorp M,Houwen S,Hartman E,et al.Effect of a ball skill intervention on children’s ball skills and cognitive functions[J].Med Sci Sports Exerc,2014,46(2):414-423.

Chen A G,Jiang R W,Ji X H,et al.Effects of 8-week moderate fancy rope skipping training on executive function in preadolescent deaf children:a school-based experimental study[J]. Journal of Sports and Science,2015,36(4):105-109.

Ziereis S,Jansen P.Effects of physical activity on executive function and motor performance in children with ADHD[J].Research in developmental disabilities,2015,38 (12):181-191.

Choi J W,Han D H,Kang K D,et al.Aerobic exercise and attention deficit hyperactivity disorder:brain research[J].Medicine and science in sports and exercise,2015,47(1):33-47.

Chen M D,Tsai H Y,Wang C C,et al.The effectiveness of racket-sport intervention on visual perception and executive functions in children with mild intellectual disabilities and borderline intellectual functioning[J].Neuropsychiatric disease and treatment,2015,11(9): 2287-2297.

Chen A G,Jin L,Zhu L N,et al.Effects of eleven-week aerobic exercises on working memory of deaf children:evidence from behavior and functional MRI[J].Chin J Sports Med,2016,35(12):1132-1139.

Pan J L,Yin H C,Chen A G,et al.An experimental study on the effect of exercise intervention on the executive functions of primary students with and without learning difficulties[J].China Sport Science,2016,36(6):84-91.

Pan J L,Yin H C,Chen A G,et al.An experimental study of the effect of exercise intervention on the executive function of primary school students with math learning difficulties[J].Chinese Journal of Special Education,2016,22(5):54-62.

Pan C Y,Chu C H,Tsai C L,et al.A racket-sport intervention improves behavioral and cognitive performance in children with attention-deficit/hyperactivity disorder[J].Research in developmental disabilities,2016,57(6):1-10.

Yin H C,Pan J L,Liu M,et al.The effects of an exercise intervention on the executive functions of primary school students with learning difficulties and normally developing primary school students:a comparison of the time course efficiency[J].Chinese Journal of Special Education,2017(6):55-62.

Liu Y,Yang N.An experimental study of the effect of orienteering exercises on the cognitive ability of children with ADHD[J].Chinese Journal of Special Education,2018,25 (11):39-44.

Chen A G,Dong X X,Zhu L N,et al.Effects of exercise intervention on executive control in deal children:a multimodal magnetic resonance imaging research[J].Journal of Sports and Science,2018,39(4):52-59.

Yin H C,Cui L,Pan J L,et al.Developmental and empirical study of exercise intervention program for improving executive function of pupils with learning difficulties[J].Journal of Wuhan Institute of Physical Education,2018,52(6):78-89.

Kadri A,Slimani M,Bragazzi N L,et al.Effect of taekwondo practice on cognitive function in adolescents with attention deficit hyperactivity disorder[J].International journal of environmental research and public health,2019,16(2):204-213.

Pan C Y,Tsai C L,Chu C H,et al.Effects of physical exercise intervention on motor skills and executive functions in children with ADHD:a pilot study[J].Journal of attention disorders,2019,23(4):384-397.

Song Y,Liu Y,Yang N,et al.Research on orienteering exercise improving the execution function of children with attention deficit hyperactivity disorder[J].Journal of Physical Education,2020,27(3):110-115.

Silva L A D,Doyenart R,Henrique Salvan P,et al.Swimming training improves mental health parameters,cognition and motor coordination in children with Attention Deficit Hyperactivity Disorder[J].International journal of environmental health research,2020,30 (5):584-592.

Tse A,Anderson D,Liu V,et al.Improving executive function of children with autism spectrum disorder through cycling skill acquisition[J].Med Sci Sport Exer,2021,53(5): 1417-1424.

Liang X,Qiu H,Wang P,et al.The impacts of a combined exercise on executive function in children with ADHD:a randomized controlled trial[J].Scandinavian Journal of Medicine & Science in Sports,2022,5(0):1-16.
